# Genome-Wide Identification of Cellular Pathways and Key Genes That Respond to Sodium Bicarbonate Stress in *Saccharomyces cerevisiae*

**DOI:** 10.3389/fmicb.2022.831973

**Published:** 2022-04-12

**Authors:** Xiuling Cao, Tingting An, Wenhao Fu, Jie Zhang, Huihui Zhao, Danqi Li, Xuejiao Jin, Beidong Liu

**Affiliations:** ^1^State Key Laboratory of Subtropical Silviculture, College of Forestry and Biotechnology, Zhejiang A&F University, Hangzhou, China; ^2^Department of Chemistry and Molecular Biology, University of Gothenburg, Gothenburg, Sweden; ^3^Center for Large-Scale Cell-Based Screening, Faculty of Science, University of Gothenburg, Gothenburg, Sweden

**Keywords:** sodium bicarbonate, *Saccharomyces cerevisiae*, genome-wide screening, NaHCO_3_, transcriptome

## Abstract

Sodium bicarbonate (NaHCO_3_) is an important inorganic salt. It is not only widely used in industrial production and daily life, but is also the main stress in alkaline saline soil. NaHCO_3_ has a strong ability to inhibit the growth of fungi in both natural environment and daily application. However, the mechanism by which fungi respond to NaHCO_3_ stress is not fully understood. To further clarify the toxic mechanisms of NaHCO_3_ stress and identify the specific cellular genes and pathways involved in NaHCO_3_ resistance, we performed genome-wide screening with NaHCO_3_ using a *Saccharomyces cerevisiae* deletion mutant library. A total of 33 deletion mutants with NaHCO_3_ sensitivity were identified. Compared with wild-type strains, these mutants had significant growth defects in the medium containing NaHCO_3_. Bioinformatics analysis found that the corresponding genes of these mutants are mainly enriched in the cell cycle, mitophagy, cell wall integrity, and signaling pathways. Further study using transcriptomic analysis showed that 309 upregulated and 233 downregulated genes were only responded to NaHCO_3_ stress, when compared with yeast transcriptomic data under alkaline and saline stress. Upregulated genes were mainly concentrated in amino acid metabolism, steroid biosynthesis, and cell wall, while downregulated genes were enriched in various cellular metabolisms. In summary, we have identified the cellular pathways and key genes that respond to NaHCO_3_ stress in the whole genome, providing resource and direction for understanding NaHCO_3_ toxicity and cellular resistance mechanisms.

## Introduction

NAHCO_3_ also known as baking soda, is often used as the main component of carbon dioxide sources in baking powder, beverages, dry powder fire extinguishers, and many other applications. It is also widely used in industrial production and the medical field. For example, NaHCO_3_ is often used as a filler for industrial production of fiber and rubber. Its medical use has gradually expanded from simple metabolic acidosis treatment to the remission and treatment of respiratory, digestive, circulatory, immune system, and cancer diseases (Choi and Kim, 2012; Di Iorio et al., 2019). However, unreasonable use can lead to cytotoxicity and environmental pollution.

In addition, NaHCO_3_ and sodium carbonate (Na_2_CO_3_) are the most common alkaline salts, which are the main stress components in saline–alkali soils (Xiao-shan et al., 2017). Worldwide, 434 million hectares soils are affected by saline–alkali stress due to the accumulation of NaHCO_3_ and Na_2_CO_3_ (Shi and Sheng, 2005; Jin et al., 2017; Xiang et al., 2019). The effects of NaHCO_3_ on the growth of plants, especially crops, have been extensively studied due to its important economic interests (Sun et al., 2016; Xiang et al., 2019; Fan et al., 2021). However, research on fungal diversity in saline–alkali land has been lacking for a long time, although it is known that fungal growth in saline–alkali land is generally inhibited (Wei and Zhang, 2018).

Moreover, there are many examples of using NaHCO_3_ to inhibit fungi, or using together with yeast in daily production and life. For example, the combine use of live yeast and NaHCO_3_ for dietary feed additives in domestic animals (Galip, 2006; Marden et al., 2008). NaHCO_3_ inhibits the production of pathogenic toxins and enhances biocontrol efficacy of antagonistic yeasts on postharvest fungal diseases (Samapundo et al., 2007; Geng et al., 2011; Letscher-Bru et al., 2013; Zhang et al., 2020). NaHCO_3_ is also widely used to formulate toothpaste, mouthwashes, and cosmetic products. There are some reports that NaHCO_3_ possesses antimicrobial activity against oral microorganisms (Bidra et al., 2011; Fu et al., 2014). Therefore, it is urgent to clarify the reasons for the influence of NaHCO_3_ on the growth of fungi and the resistance mechanism of fungi to NaHCO_3_.

*Saccharomyces cerevisiae* is a representative of fungi. It not only has high scientific research value, but also has a very broad application in production and life, and has extremely high economic value. The life regulation mechanisms in *S. cerevisiae* are homologous with and highly similar to other fungi and those multicellular organisms. *Saccharomyces cerevisiae*, however, has the advantages of a short growth cycle, clear genetic background, easy gene manipulation, and wide application, making it an important model organism for basic and applied research (Gershon and Gershon, 2000). *Saccharomyces cerevisiae* has established multiple sets of genome-wide genetic modification libraries, which are widely used in cell biology, genetics, pharmacy, and drug mechanism research (Bammert and Fostel, 2000). A large body of work has been carried out using yeast whole-genome screening. For example, Baetz et al. (2004) using whole-genome screening and found that dihydromotuporamine C can directly affect the biosynthesis of sphingolipid in yeast; McLaughlin et al. (2009) uncovered a critical role for mitochondrial translation and membrane maintenance in trichothecene toxicity through high-throughput screening of a yeast deletion mutant library; and our lab used whole-genome screening to characterize how oxidative phosphorylation plays a critical role in cellular tolerance to lithium hexafluorophosphate (Jin et al., 2021).

At present, a large number of studies have been carried out in yeast on the response of osmotic, oxidative, and alkaline pH stress (Thorpe et al., 2004; Melamed et al., 2008; Serra-Cardona et al., 2015; Liang et al., 2018). However, the response and resistance mechanisms of yeast under NaHCO_3_ stress are unclear; the damage caused by NaHCO_3_ is more serious and complex, and the mechanism of its stress signal perception, transduction, and resistance warrants clarification. Identifying the genes that respond to NaHCO_3_ stress can aid in the understanding of the genetic basis of resistance to saline–alkali stress and can provide valuable information for guiding scientific use in daily life and improving the organism’s resistance. The aim of the present study is to reveal the key genes and cellular pathways of cellular resistance to NaHCO_3_, at the genomic level, in *S. cerevisiae*.

## Materials and Methods

### Yeast Strain and Culture Conditions

All strains and mutants in this study are based on the *S. cerevisiae* strain BY4741 (*MATa; his3Δ-1; leu2Δ-0; met15Δ-0;* and *ura3Δ-0*). The collection analyzed here, called the SGA-V2 collection, contains about 4,200 mutants (with mutations in non-essential genes), and was a gift provided by Prof. Charles Boone (University of Toronto, Canada; Tong et al., 2001, 2004). Mutant *his3Δ::KanR* in this collection was designed as the control strain and added as a border around four edges of each plate (Tong and Boone, 2007). The strains were grown at 30°C, in YPD medium (1% yeast extract, 2% peptone, and 2% glucose) with 200 mg·L^−1^ G418 (YPD + G418). NaHCO_3_-containing plates were made with YPD agar medium that was supplemented with 40 mM NaHCO_3_ and 200 mg·L^−1^ G418. For the yeast growth curve, the optical density at 600 nm (OD_600_) absorbance value of yeast BY4741 under different NaHCO_3_ concentration treatments was measured using Ultrospec 2100 pro (Biochrom, United Kingdom) spectrophotometer. The measurement was performed every 2 h and the growth curve was drawn.

### Genome-Wide NaHCO_3_ Screen

The collection of yeast gene deletion mutants was arrayed in 384 formats. Firstly, the strains, in 384-well frozen stock plates, were spotted onto YPD agar plates with G418 added (YPD + G418 plates) using the 384-pining replicator operated by Singer Rotor (Singer Instruments, United Kingdom), and the plates were incubated at 30°C for 3 days. Then, the 384-clone array in each plate was replicated in quadruplicate to a new plate containing either no NaHCO_3_ or 40 mM NaHCO_3_ to generate a 1,536-density array, with four replications per plate for each strain. These array plates were incubated for 2 days at 30°C before imaging with PhenoBooth (Singer Instruments, United Kindom). SGAtools^1^ (Wagih et al., 2013) was used to evaluate the growth of individual strains with or without NaHCO_3_ treatment. The score for each strain was calculated based on standardized colony size for control and NaHCO_3_ treatment. Mutants that were sensitive to NaHCO_3_ (with a cutoff of less than −0.3) were selected, according to previous experimental studies (Babazadeh et al., 2019). This value was chosen because a score value lower than −0.3 usually indicates a relatively strong inhibitory effect, and most of the colonies screened with a score value lower than −0.3 can be confirmed by other methods. This experiment was performed in three independent replicates.

### Spot Tests Analysis

Adjust the strains cultured overnight to an OD_600_ = 0.1 and cultured in YPD + G418 liquid medium at 30°C until reaching OD_600_ = 0.5. Then, cultures were serially diluted in a 10-fold gradient and spotted on plates with different treatments. After 48 h incubation at 30°C, yeast growth was imaged and analyzed.

### Data Processing and Bioinformatics Analysis

Candidate hits were analyzed for enrichment in the Gene Ontology (GO) and Kyoto Encyclopedia of Genes and Genomes (KEGG) databases by comparing with corresponding background set of genes, using ClueGO in Cytoscape with a cutoff of *p* < 0.05. The functional classification analysis was based on the functional description from the Saccharomyces Genome Database.^2^ The location distribution of selected genes associated with NaHCO_3_ sensitivity was determined through the Yeast GFP Fusion localization database^3^ (Huh et al., 2003). Venn diagram analysis was performed by using Venny 2.1.^4^

### Transcriptional RNA Sequence Analysis

Dilute overnight cultured saturated yeast cells into fresh YPD liquid medium to OD_600_ = 0.1. At this time, NaHCO_3_ was added to the experimental group to make the final concentration of 40 mM, while the control group was not added. Harvest cells grown at 200 rpm, 30°C to OD_600_ = 0.85 for transcriptome sequencing, with four sample replicates making up the set for each treatment. Total RNA was extracted using TRIzol reagent (Thermo Fisher, 15,596,018), and total RNA quantity and purity were analyzed using a Bioanalyzer 2100 and an RNA 6000 Nano LabChip Kit (Agilent, CA, United States, 5,067–1,511). High-quality RNA reads with an RNA integrity number >7.0 were used to construct cDNA library for sequencing. The cDNAs in the library were sequenced on an Illumina NovaseqTM 6000 platform to generate 2 × 150 bp paired-end reads. Cutadapt was used to remove the Illumina adapter contamination and for trimming the reads and clipping the low-quality bases. After this processing, at least 5 Gb of clean reads were obtained. Gene differential expression analysis was performed by DESeq2 software between two different groups. The genes with the parameter of false discovery rate below 0.05 and absolute fold change ≥2 were considered to represent differentially expressed genes (Love et al., 2014).

## Results

### The Genome-Wide Screen Identified Deletion Mutants With Increased Sensitivity to NaHCO_3_

**Figure 1:**
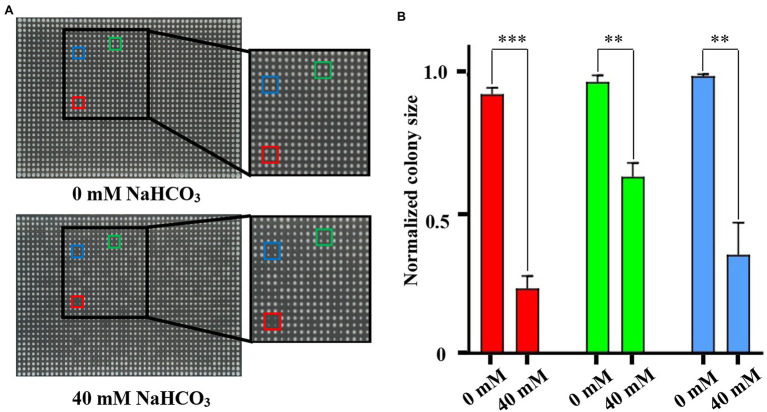
Identification of appropriate NaHCO_3_ concentration for genome-wide screening using plate SGA-V2-2. **(A)** The different colored boxes frame the growth of the same mutant on 0 and 40 mM NaHCO_3_, respectively. (Red: *rvs161Δ*; Green: *ste50Δ*; and Blue: *pho2Δ*). Compared with the control, the same strains in the experimental group of 40 mM NaHCO_3_ showed obvious growth defects. **(B)** The colony sizes of the framed strains in **(A)** were analyzed. Error bars indicate standard error. **p < 0.01; ***p < 0.001; Student’s *t*-test.

NAHCO_3_ is an important material in the field of industrial production, food, and drug additives. Although NaHCO_3_ is widely used for fungal inhibition, few studies were carried out on its inhibitory mechanism. In order to improve our ability to use NaHCO_3_ scientifically, we must improve our understanding of the intracellular actions and pathways of NaHCO_3_ and the mechanisms of cell response to NaHCO_3_ stress. Toward this end, we performed genome-wide screening of genes responding to NaHCO_3_ stress using the SGA-V2 library of *S. cerevisiae* single-gene deletion mutants (Tong et al., 2001, 2004). Firstly, the concentration of NaHCO_3_ for genome-wide screening was determined. For this, we randomly selected the culture plate designated SGA-V2-2. Each 384-arrayed mutant group was replicated in quadruplicate to yield a 1,536 array, using a Singer Rotor, and the strains in the 1,536-array were cultivated on culture plates containing different concentrations of NaHCO_3_. The plates were cultured at 30°C for 48 h and then photographed. The concentration at which the growth size of the wild-type strain in the outermost two rows of the culture plate was reduced by about 1/3 to 1/2, compared with the growth state of the wild-type strain on the 0 mM culture plate, was selected for screening. Using this process, we determined that the concentration of 40 mM NaHCO_3_ would be an appropriate concentration for genome-wide screening (Figure 1). The growth phenotypes of the mutants that were sensitive to NaHCO_3_ differed significantly between the control and experimental plates. Taking the SGA-V2-2 culture plate as an example, compared with the control group, the sensitive strain showed significant growth defects in the experimental group treated with 40 mM NaHCO_3_ (Figure 1).

We screened the entire collection (about 4,200 gene deletion mutants) on YPD + G418 agar plates containing 0 or 40 mM NaHCO_3_. These deletion mutants were distributed in 14 culture plates. The culture plates were incubated at 30°C for 48 h, and the images of the control plates and experimental plates were collected by PhenoBooth and uploaded to the SGAtools website for quantification and analysis. On the basis of three independent repeated tests, with screening criteria set to score value from the SGAtools website of three times less than −0.3 and the *p* value of less than 0.05, we confirmed that 69 deletion mutants showed sensitivity to NaHCO_3_ (Supplementary Table S1). Compared with the control group, the colony size of these sensitive deletion mutant strains showed a significant reduction, indicating that 40 mM NaHCO_3_ has a significant inhibitory effect on them.

### Spot Test to Verify Screening Results

**Figure 2:**
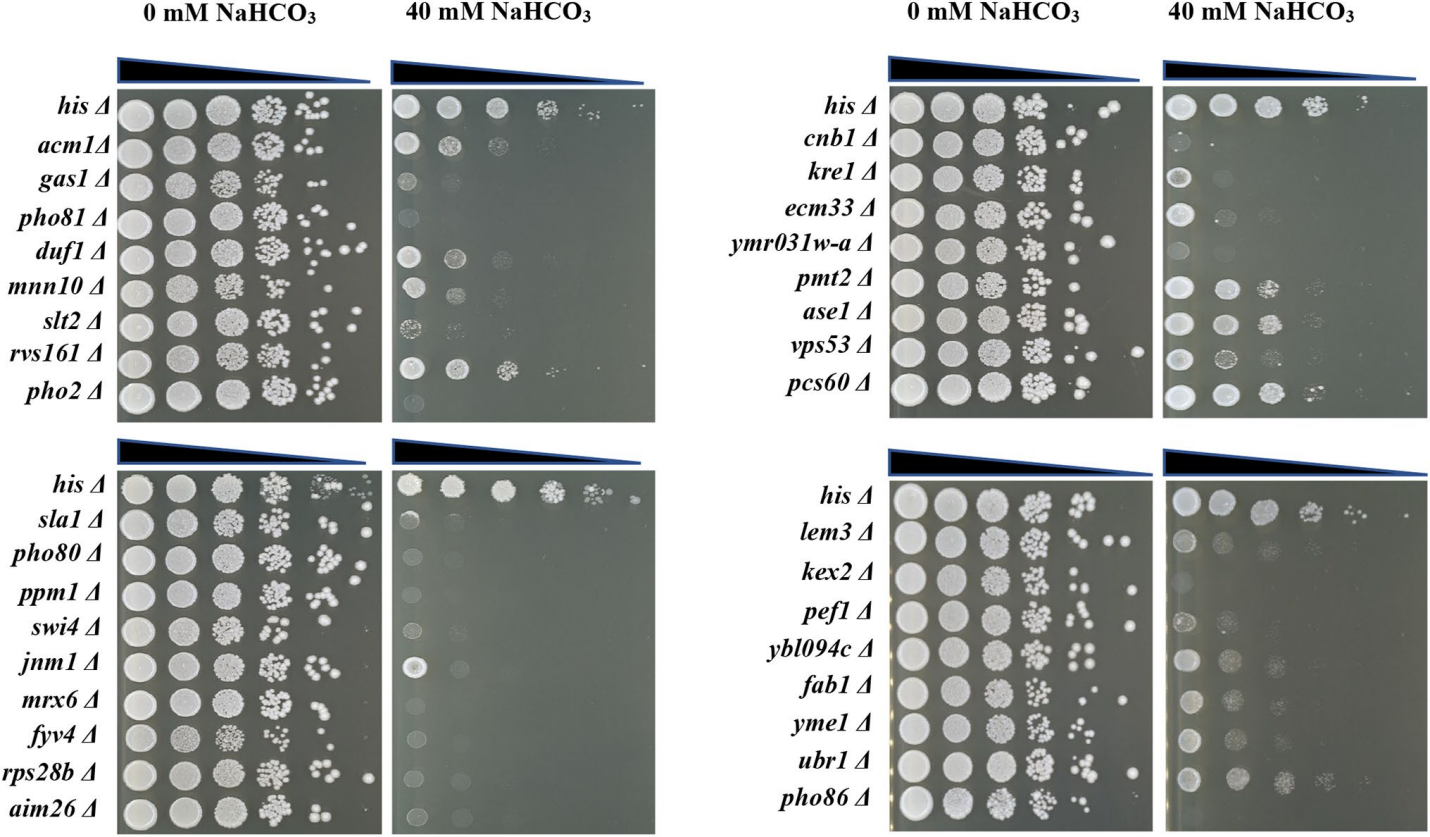
Spot test of the 33 selected deletion mutants. The control strain and deletion mutants were grown to mid-log phase in YPD + G418 liquid medium and then diluted to an OD_600_ = 0.5. Each strain was serially diluted in a 10-fold gradient and 5 μl were spotted onto YPD + G418 agar plates either containing 0 mM NaHCO_3_ or 40 mM NaHCO_3_ and incubated at 30°C. Plates were photographed after 48 h.

In order to verify the accuracy of the genome-wide screening results, the drop test experiment was performed to verify the growth phenotypes of the 69 selected deletion mutants. As shown in Figure 2, 33 of the 69 deletion mutants display enhanced growth defect when exposed to 40 mM NaHCO_3_ compared to the control strain, although they were inhibited to varying degrees, implying the importance of the 33 genes deleted in sensitive mutants in NaHCO_3_ resistance. The information of the 33 mutants together with their quantitative fitness scores is summarized in Table 1. Among these, 16 mutants barely grew on the plates with 40 mM NaHCO_3_, including *pho86Δ*, *pho80Δ*, *pho81Δ*, and *pho2Δ*, which were deficient in genes related to phosphate metabolism and the cell cycle, as well as *kex2Δ*, *ymr031w-aΔ*, *cnb1Δ*, *gas1Δ*, *slt2Δ*, *sla1Δ*, *ppm1Δ*, *swi4Δ*, *fyv4Δ*, *rps28bΔ*, *mrx6Δ*, and *aim26Δ*, which were deficient in genes playing important roles in the process of cell metabolism and maintenance of the normal growth state. Therefore, since many genes were identified to be involved in NaHCO_3_ resistance, the inhibitory effect of NaHCO_3_ is not achieved through a single pathway, but may involve a variety of cellular responses. Previous studies have revealed a set of multidrug-resistant genes which are required for a large multitude of stresses. Our deletion mutant array contains 14 genes characterized previously as contributors to general multidrug resistance, including *GAS1*, *MNN10*, *SLT2*, *RVS161*, *SLA1*, *PHO80*, *KRE1*, *CNB1*, *PMT2*, *LEM3*, *KEX2*, *FAB1*, *ECM33*, and *YME1* (Parsons et al., 2004, 2006; Hillenmeyer et al., 2008). In addition, some genes with unknown function, such as *DUF1*, *AIM26*, *YBL094C*, and *YMR031W-A*, were also revealed through the screening to be involved. At present, the functions of these genes in resistance to NaHCO_3_ have not been fully elaborated. Knowledge of these functions would be helpful in exploring the new mechanism implied by these results.

**Table 1:** Genome-wide screening results of 33 selected NaHCO_3_-sensitive mutants.

Gene	ORF	Score1^a^	*p* value1^a^	Score2^a^	*p* value2^a^	Score3^a^	*p* value3^a^
ACM1	YPL267W	−0.37095	0.00001	−0.66948	0.00005	−0.5414	0.00005
GAS1	YMR307W	−0.49761	0.00015	−0.48975	0.00015	−0.54929	0.00015
PHO81	YGR233C	−0.58694	0	−0.4714	0.00003	−0.44746	0.00003
DUF1	YOL087C	−0.47937	0.00006	−0.41218	0.00004	−0.60865	0.00014
MNN10	YDR245W	−0.51543	0.00096	−0.46468	0.00194	−0.51503	0.00096
SLT2	YHR030C	−0.78787	0.00023	−0.84749	0.00053	−0.6906	0.00053
RVS161	YCR009C	−0.73636	0.00133	−0.74711	0.00002	−0.81866	0.00002
PHO2	YDL106C	−0.53138	0.00004	−0.72423	0.00025	−0.7015	0.00025
CNB1	YKL190W	−0.52263	0.00002	−0.57064	0.00008	−0.55943	0.00008
KRE1	YNL322C	−0.47556	0.00009	−0.47792	0.00009	−0.44205	0.00009
ECM33	YBR078W	−0.50875	0.00002	−0.43931	0.00002	−0.39144	0.00002
YMR031W-A	YMR031W-A	−0.64322	0.00193	−0.45696	0.00007	−0.32843	0.00007
SLA1	YBL007C	−0.4349	0.00002	−0.49435	0.00013	−0.42361	0.00002
PHO80	YOL001W	−0.46344	0.00002	−0.5348	0.0002	−0.73544	0.00023
PPM1	YDR435C	−0.4195	0.00004	−0.3638	0.00001	−0.34115	0.00001
SWI4	YER111C	−0.3583	0.00002	−0.34755	0.00005	−0.30362	0.00005
PMT2	YAL023C	−0.46166	0.00004	−0.50745	0.00036	−0.41833	0.00004
RPS28B	YLR264W	−0.39336	0.00001	−0.38226	0.00009	−0.42705	0.00001
AIM26	YKL037W	−0.33889	0.00016	−0.41739	0	−0.42533	0
JNM1	YMR294W	−0.4497	0	−0.45956	0	−0.45743	0
MRX6	YNL295W	−0.35736	0.00006	−0.37911	0.00006	−0.36193	0.00006
LEM3	YNL323W	−0.30015	0	−0.32	0	−0.3371	0
KEX2	YNL238W	−0.40498	0.00001	−0.42015	0.00001	−0.38429	0.00001
PEF1	YGR058W	−0.32749	0.00006	−0.31093	0.00005	−0.34778	0.00006
UBR1	YGR184C	−0.4068	0.00004	−0.42734	0.00131	−0.34517	0.00131
FYV4	YHR059W	−0.41197	0.00002	−0.40458	0.00019	−0.30176	0.00019
YME1	YPR024W	−0.4389	0.00017	−0.36472	0.00033	−0.36019	0.00033
PHO86	YJL117W	−0.34046	0.00014	−0.40671	0.00002	−0.44002	0.00002
YBL094C	YBL094C	−0.32554	0.0033	−0.50264	0.00116	−0.38208	0.0033
FAB1	YFR019W	−0.48358	0.00017	−0.55748	0.00012	−0.49166	0.00017
ASE1	YOR058C	−0.42186	0	−0.49015	0.00027	−0.80064	0.00091
VPS53	YJL029C	−0.32385	0	−0.31397	0	−0.31601	0
PCS60	YBR222C	−0.44795	0.00008	−0.44396	0.00015	−0.41986	0.00008

a *1, 2, and 3 indicate three independent replicates*.

### Classification of Genes Related to NaHCO_3_ Resistance

**Figure 3:**
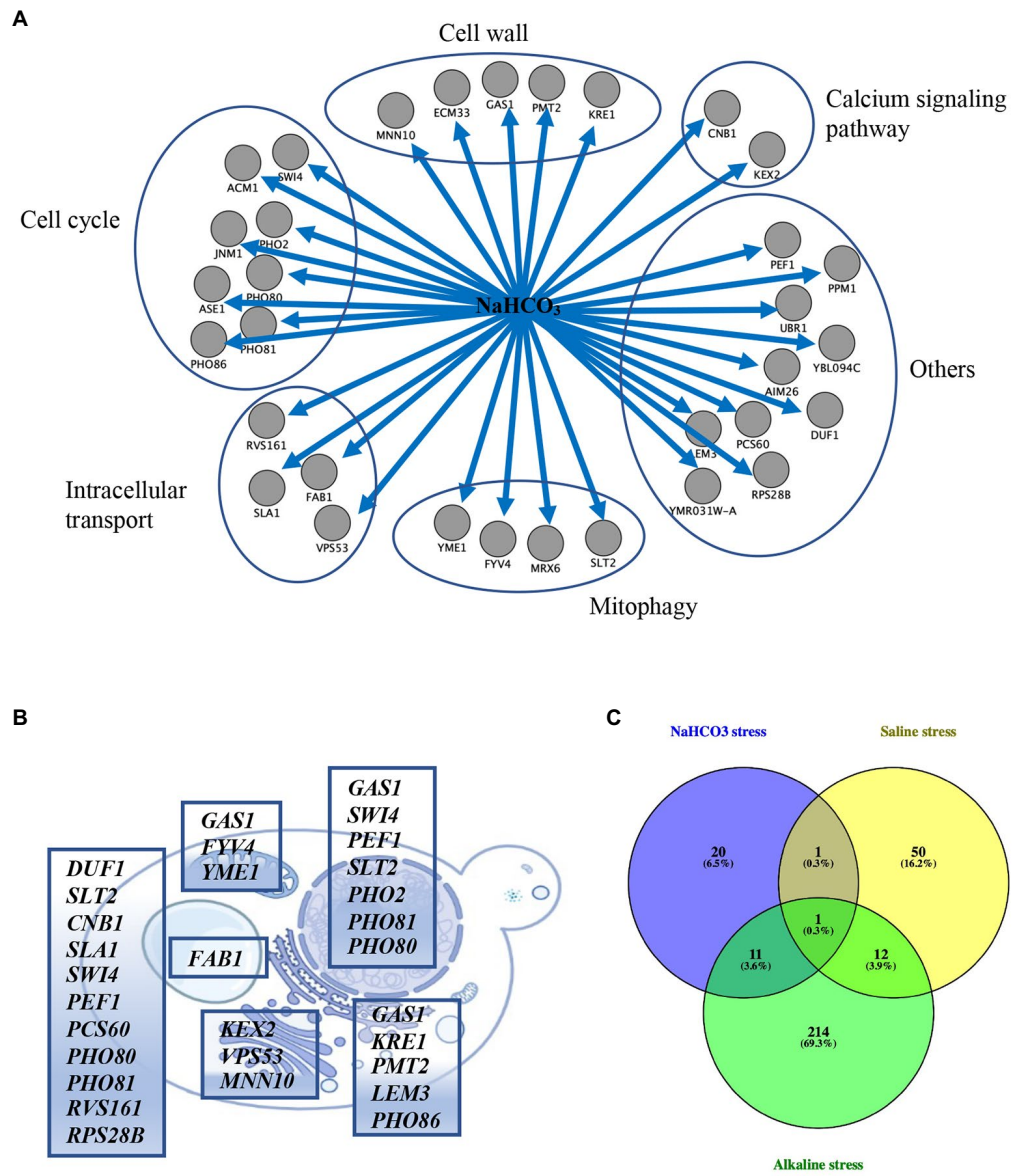
Enrichment and localization analysis of the 33 selected genes. **(A)** Functional classification of the 33 genes. **(B)** Cellular localization of the 33 selected genes. These genes are distributed in the cytoplasm, nucleus, Golgi, vacuole, endoplasmic reticulum, and mitochondrion. **(C)** Venn diagram analysis of the 33 selected genes vs. the 238 and 64 genes whose deletion results in growth defects under alkaline and saline stress, respectively.

To further analyze the functions and pathways of the genes involved in NaHCO_3_ responses, functional classification of the 33 genes was performed according to the functional description from the Saccharomyces Genome Database.^5^ Most of the genes were placed into specific functional categories, including cell cycle, cell wall, calcium signaling pathway, intracellular transport, and mitophagy (Figure 3A). Cellular localization analysis showed that *DUF1*, *CNB1*, *SLA1*, *PCS60*, *RVS161*, and *RPS28B* were localized in the cytoplasm; *FAB1* was localized in vacuoles; *FYV4* and *YME1* were localized in mitochondria; *KRE1*, *PMT2*, *LEM3*, and *PHO86* were localized in the endoplasmic reticulum (ER); *PHO2* was localized in the nucleus; and *SWI4*, *PEF1*, *SLT2*, *PHO80*, and *PHO81* were localized in both the cytoplasm and the nucleus (Figure 3B). Although *PHO2* is also involved in phosphate metabolism as *PHO80* and *PHO81*, it might only play a role in the nucleus. *GAS1* participates in the biosynthesis and morphogenesis of the cell wall and is distributed in the nucleus, mitochondria, and ER (Figure 3B). Together, it suggested that NaHCO_3_ has widespread effects on the yeast physiology, and the integrity of these cellular processes plays a key role in the response of cells to NaHCO_3_ stress.

Previous studies have determined the genes in yeast whose deletion result in alkaline pH sensitivity and salt sensitivity through conventional genetic screens (Giaever et al., 2002; Serrano et al., 2004). In order to analysis whether the 33 genes we identified are specifically response to NaHCO_3_ or are involved in general stress response, we compared the 33 mutants with the 238 mutants showing defective growth under alkaline stress yielded from previous studies (Giaever et al., 2002; Serrano et al., 2004; Loha et al., 2018). Twelve genes were identified as overlapping (Figure 3C; Supplementary Table S2), including *GAS1*, *PHO80*, *PHO81*, *PHO2*, *PHO86*, *SLT2*, *CNB1*, *KRE1*, *SWI4*, *AIM26*, *KEX2*, and *FAB1*. Likewise, analysis of the set of genes induced by both salt stress and NaHCO_3_ exposure yielded two genes (Giaever et al., 2002; Figure 3C; Supplementary Table S2), including *CNB1* and *PEF1*. These overlapped genes indicated that cells might share overlapping mechanisms of resistance to different kinds of stresses.

### NaHCO_3_ Stress on Yeast Are Beyond the Combination of High pH and Salt

**Figure 4:**
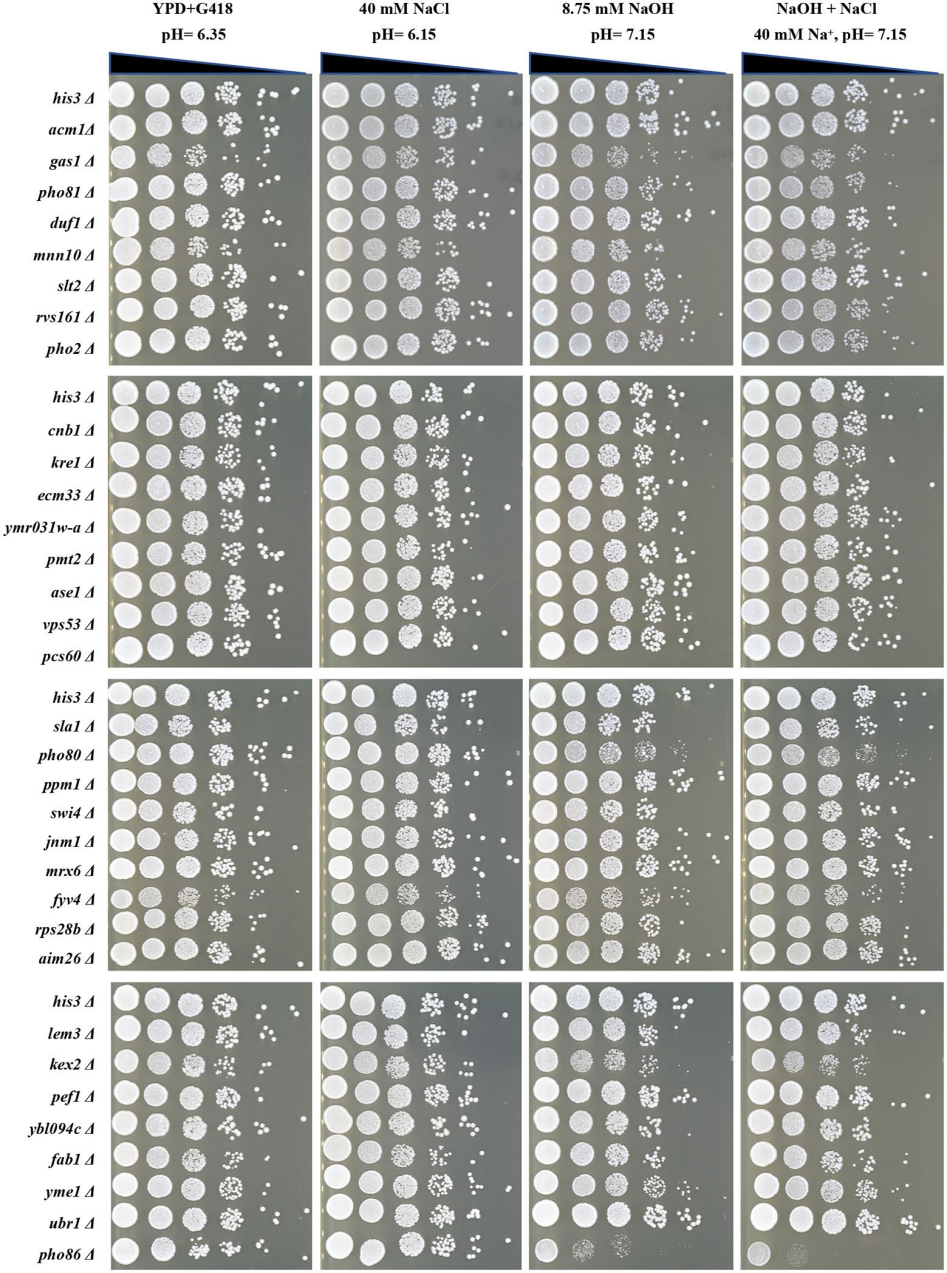
Spot test of the 33 selected deletion mutants under stresses with the same pH and Na^+^ concentration as 40 mM NaHCO_3_. The control strain and deletion mutants were grown to mid-log phase in YPD + G418 liquid medium and then diluted to an OD_600_ = 0.5. Each strain was serially diluted in a 10-fold gradient and 5 μl were spotted onto differently treated agar plates. The pH = 7.15 plates were adjusted with NaOH. Plates were incubated at 30°C and photographed after 48 h.

In order to reveal whether toxic effects of NaHCO_3_ is a combination of mechanisms coming from high pH and salt, we measured the pH of the medium under different stresses. The final pH of YPD medium is 6.35 under normal conditions. This is consistent with the fact that yeast prefers an acidic external pH to maintain proton gradient over the plasma membrane. However, the medium containing 40 mM NaHCO_3_ used in our study has a pH of 7.15, which, although close to neutral pH, is more alkaline than that under normal conditions. Then, the 33 mutants were tested for growth on plates containing 40 mM NaCl or at pH 7.15, respectively. These mutants were found to be not significantly sensitive to medium with the same concentration of Na^+^ (40 mM NaCl; Figure 4), indicating that effects of Na^+^ in NaHCO_3_ stress are limited to a small proportion. When exposed to media at pH 7.15, *gas1Δ*, *kex2Δ*, *pho80Δ*, and *pho86Δ* exhibit defective growth, while other mutants did not show significant differences (Figure 4). This is consistent with previous reports that these four genes are essential for the response to high pH (Giaever et al., 2002; Serrano et al., 2004; Loha et al., 2018). Other reported alkaline-sensitive mutants were not validated in our drop test experiments, probably because the pH we used was close to neutral rather than alkaline. Therefore, although NaHCO_3_ stress could induce the growth defect of certain alkaline-sensitive mutants, the toxic effect of NaHCO_3_ was more profound than that of NaOH at the same pH.

We also conducted an experimental group that exposed to NaOH and NaCl at the same time, allowing the concentration of Na^+^ to 40 mM, and the pH to 7.15, the same Na^+^ concentration and pH as 40 mM NaHCO_3_. However, a synergistic enhancing effect between NaCl and NaOH stress was not observed in this study. Among 33 mutants, only *gas1Δ*, *kex2Δ*, *pho80Δ*, and *pho86Δ* exhibited significantly decreased sensitivity, while others did not (Figure 4). This implies that NaHCO_3_ has a more specific mechanism of toxicity than the combination of salt stress and alkali stress, although cells may share overlapping control mechanisms for resistance to both alkaline stress and NaHCO_3_ stress.

### Global Transcriptional Changes in Response to NaHCO_3_ Stress

**Figure 5:**
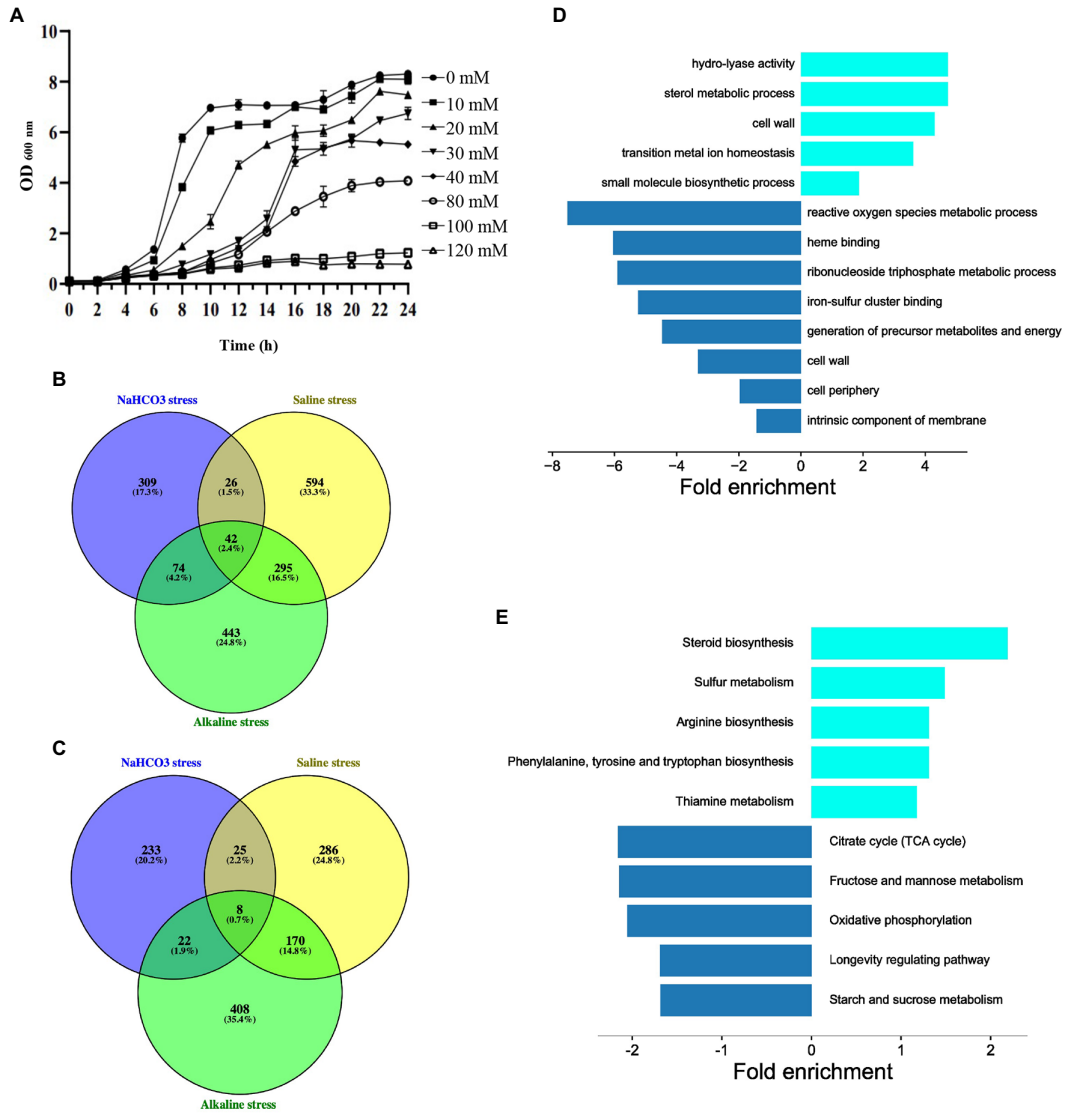
Transcriptional analysis of gene expression under NaHCO_3_ treatment. **(A)** Growth curve of BY4741 under different concentrations of NaHCO_3_. **(B)** Venn diagram analysis of 451 upregulated genes under NaHCO_3_ stress vs. the 854 and 957 genes which also are upregulated under alkaline and saline stress, respectively. **(C)** Venn diagram analysis of 288 downregulated genes under NaHCO_3_ stress vs. the 608 and 489 genes, which are also downregulated under alkaline and saline stress, respectively. **(D)** GO enrichment analysis of 309 upregulated and 233 downregulated genes that only respond to NaHCO_3_ stress. **(E)** KEGG enrichment analysis of 309 upregulated and 233 downregulated genes that only respond to NaHCO_3_ stress.

In order to further explore the mechanism of cells responding to NaHCO_3_, we conducted transcriptomic analysis of the changes in genes under NaHCO_3_ stress. We first measured the growth curve of yeast at different NaHCO_3_ concentrations. It can be seen that as the concentration of NaHCO_3_ increased, the growth of the yeast was significantly inhibited (Figure 5A). Among the different concentrations tested, 40 mM NaHCO_3_ was considered the most suitable condition. At this concentration, cell growth was significantly inhibited, but with the extension of culture time, the cells could still reach a certain growth density. After 10 h of growth under the condition of 40 mM NaHCO_3_, the cells began to enter the early phase of the logarithmic growth phase. At this time, the effect of NaHCO_3_ on the cells should have been reflected at the transcription level. Thus, the gene expression changes were analysis under the condition of 40 mM NaHCO_3_.

Whole transcriptome sequencing revealed that, compared with the control group, after 40 mM NaHCO_3_ treatment, the expression levels of 451 genes were significantly upregulated, and the expression levels of 288 genes were significantly downregulated (Supplementary Table S3). Then, correlation estimate for the 33 NaHCO_3_-resistant genes tested in both genome-wide screen and transcriptomic analysis was performed to verify whether the expression of the genes required for growth is also significantly increased. However, these 33 genes were unexpectedly either downregulated or unchanged. We speculate that these genes may function through post-translational modifications, or their downregulation may be part of a general downregulation effect on metabolism, associated with slow cell growth.

In order to identify the genes specifically response to NaHCO_3_ stress, we compared our results with different sets of data regarding the transcriptional response to saline stress and alkaline stress (Posas et al., 2000; Rep et al., 2000; Lamb et al., 2001; Yale and Bohnert, 2001; Serrano et al., 2002, 2006; Viladevall et al., 2004; Melamed et al., 2008; Halbeisen and Gerber, 2009; Casado et al., 2011; Casamayor et al., 2012). Around 142 genes upregulated under our condition can be also induced by either saline stress or alkaline stress (Figure 5B; Supplementary Table S4), while 55 genes downregulated under NaHCO_3_ overlapped with those under either saline stress or alkaline stress (Figure 5C; Supplementary Table S4). These genes may be involved in the general stress response, especially the overlapping genes that are changed under all three stress conditions. Although we cannot rule out the possibility that the remaining 309 upregulated and 233 downregulated genes would display expression changes under other types of stress, such as heat shock, to some extent, it provides a set of key genes for further study of how cells are respond to NaHCO_3_.

In order to further identify the functional classification of the 309 upregulated and 233 downregulated genes, GO and KEGG enrichment analysis on these genes were performed. Upregulated genes were mainly concentrated in amino acid metabolism, steroid biosynthesis, and cell wall, while downregulated genes were enriched in various cellular metabolisms (Figures 5D,E). It has been demonstrated that under stress, cells tend to optimize cellular resources for stress adaptation, allowing the massive expression of genes involved in stress adaptation, while shutting down the expression of genes involved in proliferation and cell cycle progression (Giaever et al., 2002). Thus, it is reasonable that genes in various cellular metabolisms are downregulated. Although in our study, significantly upregulated genes have poor correlation with their importance in growth fitness, it helps to some extent in our understanding of how cells respond to NaHCO_3_.

## Discussion

### NaHCO_3_ Has a Specific Mechanism of Action Beyond High pH and Salinity

In order to verify whether NaHCO_3_ has the characteristics of saline stress and alkaline stress, we compared the conditions of yeast external medium under different stresses. About 40 mM NaHCO_3_ results in an increase in external pH. Although the pH has increased, it is still lower than the pH that researchers usually use to identify alkaline-sensitive mutants (Giaever et al., 2002; Serrano et al., 2004). In addition, most of the 33 mutants lost their sensitivity on NaOH plates with the same pH. Therefore, NaHCO_3_ must have its own toxicity mechanisms in yeast that different from the alkalinization. Moreover, all 33 NaHCO_3_-sensitive mutants exhibited similar growth to the control strain when exposed to 40 mM NaCl (same molar quantity of Na^+^ as 40 mM NaHCO_3_), indicating that the influence of Na^+^ in NaHCO_3_ stress is much weaker. It is reasonable that yeast cells have limited responses to 40 mM Na^+^ since yeast is highly tolerant to Na^+^ (Rep et al., 2000; Yale and Bohnert, 2001). More importantly, when yeast cells were exposed to both NaOH and NaCl (40 mM Na^+^, pH 7.15), the effect of stress on NaHCO_3_-sensitive mutants was unexpectedly less. These results suggest that NaHCO_3_ has a distinct cytotoxicity mechanism in yeast.

### Common and Specific Genes Regulate Yeast NaHCO_3_ Sensitivity

Through genome-wide screen and spot test experiment, 33 genes were identified in this study to be important for NaHCO_3_ resistance. They are mainly enriched in cell wall, calcium signaling, mitophagy, intracellular transport, and cell cycle pathways. Previous studies have identified 238 alkaline-resistant genes and 64 saline-resistant genes in yeast. But only 12 genes and two genes were overlapped with those in the NaHCO_3_ screen (Figure 3C). This again implies that NaHCO_3_ may has a specific mode of action and that the cellular responses to NaHCO_3_ differ from both saline stress and alkaline stress, despite several shared genes were identified.

The 12 genes yielded in both alkaline stress and NaHCO_3_ exposure include *GAS1*, *PHO80*, *PHO81*, *PHO2*, *PHO86*, *SLT2*, *CNB1*, *KRE1*, *SWI4*, *AIM26*, *KEX2*, and *FAB1*. Among them, *KRE1* and *PMT2* are involved in 1,6-β-D-glucan and mannose type O-glycan biosynthesis, respectively, and *GAS1* encodes β-1,3-glucanosyltransferase (Breinig et al., 2004; de Medina-Redondo et al., 2010; Castells-Ballester et al., 2019). They are required for cell wall synthesis and organization. Therefore, we speculate that NaHCO_3_ might cause damage to the yeast cell wall and result in the activation of cell surface stressors, which then activate a set of effectors that regulate downstream signaling pathways. This can be confirmed by the enhanced sensitivity to NaHCO_3_ induced by *SLT2* deletion (Figure 2). Slt2 is a component of the MAPK cascade involved in maintaining of cell integrity. There is evidence that the Slt2-regulated MAPK pathway plays important roles in the adaptive response to alkaline stress, the activation of which is largely dependent on the Wsc1 cell surface sensor (Serrano et al., 2006). Therefore, yeast cells may respond to NaHCO_3_ by activating the same Slt2 MAPK pathway as alkaline stress through cell wall remodeling.

Calcium-activated phosphatase calcineurin is another signaling pathway that integrated into the alkaline stress response mechanism (Serra-Cardona et al., 2015). Cnb1 is the regulatory subunit of the yeast calcium-activated Ser/Thr protein phosphatase calcineurin (Cyert, 2003), and yeast cells under both alkaline stress and saline stress require this gene to maintain fitness. Deletion of *CNB1* also results in poor growth under NaHCO_3_ stress (Figure 2). This means that the calcium-activated phosphatase calcineurin is also essential for dealing with NaHCO_3_. In addition to the two signaling pathways discussed above, the Rim101/PacC pathway is responsible for a set of alkali-induced responses (Serra-Cardona et al., 2015). However, in our screen, *RIM101* deletion did not show significant growth changes in 40 mM NaHCO_3_. This pathway may not affect the yeast survival capability under NaHCO_3_ stress.

Evidence indicates that alkalinization also perturbs nutrient uptake of cells, such as phosphate, and it can result in the activation of phosphate acquisition-related genes. Thus, exposure to alkali stress may mimic the situation of phosphate starvation (Ariño, 2010). Genes associated with phosphate starvation responses were also identified in our screen, including *PHO2*, *PHO80*, *PHO81*, and *PHO86*. Therefore, we speculate that NaHCO_3_ may have a negative effect on nutrient homeostasis in yeast, as does alkaline stress.

Mitochondria are essential organelles that produce most of the cellular energy, the effective performance of mitochondrial function is essential for optimal cell growth under aerobic conditions. At the same time, however, mitochondria also produce reactive oxygen species that are harmful to cell physiology. Therefore, it is necessary to efficiently clean up damaged and superfluous mitochondria. Appropriate mitophagy is critical for mitochondria and cellular homeostasis. Yeast mitochondrial i-AAA protease Yme1 functions in mitochondrial quality control system by degrading unfolded or misfolded mitochondrial proteins (Weber et al., 1996), and its activity is required for mitophagy (Wang et al., 2013). In our study, *YME1* deletion significantly inhibited cell growth under NaHCO_3_ stress (Figure 2), which was not observed in previous studies of responses to saline and alkaline stress; thus, the induction of mitophagy may be a specific response to NaHCO_3_ and the normal operation of mitophagy under the influence of NaHCO_3_ may play an important role in maintaining cell homeostasis. Previous studies have reported that mitophagy in yeast requires the Slt2 MAPK pathway, thus, *SLT2* deletion would also affect mitophagy under NaHCO_3_ stress and lead to sensitive phenotype.

### Cross-Stress Adaptation Strategies Revealed by Transcriptomic Analysis

In our transcriptomic analysis, amino acid metabolism- and most cell wall-related genes were upregulated. Evidence has found that in *Puccinellia tenuiflora*, amino acid metabolism pathways are also significantly upregulated under the stress of a salt–alkali environment rich in NaHCO_3_ (Ye et al., 2019). In addition, cells can also regulate cell wall-related gene expression in the face of stress, changing the cell wall structure to actively respond to environmental changes. Extracellular proteins and receptor-like kinases on the cell membrane can recognize the changed cell wall structure and transmit environmental stress signals to the cell to activate the corresponding signal pathways (Zhao et al., 2018). Salt damage, drought stress, and other osmotic stresses can increase the expression of expandin- and xyloglucan-modifying enzyme genes to reshape the cell wall, also leading to the loss of cell wall Ca^2+^ and other cell wall changes (Tenhaken, 2014). Indeed, in previous study of yeast genomic expression programs in response to environmental changes, amino acid metabolism- and cell wall metabolism-related genes are environmental stress response genes, and thus, up- or downregulation of these genes is a common response to most of the stressful conditions (Gasch et al., 2000; Causton et al., 2001). Yeast utilizes cross-stress adaptation strategies to cope with diverse stressors. Although the genes that were significantly upregulated in the transcriptomic analysis were poorly correlated with their importance in growth fitness, the results of transcriptome extend our understanding of yeast NaHCO_3_ responses by providing a set of key genes for further investigation.

In summary, *S. cerevisiae* is a model system for the study of fungi and other life. Genome-wide screening is a good strategy to uncover key genes/proteins or functions that allow cells to cope with toxicity. Here, we present high-throughput screening results in a yeast single-gene deletion collection for sensitivity to NaHCO_3_, and a transcriptomic analysis of the changes in genes under NaHCO_3_ stress. We screened and confirmed yeast genes that play a key role in responding to NaHCO_3_ stress, and compared them to genes that play roles in other stressors to find specific genes and pathways. Our present study provides a direction to understand the response mechanism of yeast cells to NaHCO_3_.

## Data Availability Statement

The datasets presented in this study can be found in online repositories. The names of the repository/repositories and accession number(s) can be found at: Sequence Read Archive (SRA), PRJNA808812.

## Author Contributions

BL, XJ, and XC designed the experiments and revised the manuscript. XC, TA, and WF performed the experiments. XC, TA, WF, JZ, HZ, and DL analyzed the data. XC and TA mapped all the figures and wrote the manuscript. All authors contributed to the article and approved the submitted version.

## Funding

This research was funded by grants from the National Natural Science Foundation of China (32000387 and 31800163) to XC and XJ, and Zhejiang Provincial Natural Science Foundation of China (LQ19C070001) to XC, as well as the Swedish Cancer Fund (Cancerfonden; CAN 2017/643 and 19 0069) and the Swedish Natural Research Council (Vetenskapsrådet; VR 2015-04984 and VR 2019-03604) to BL.

## Conflict of Interest

The authors declare that the research was conducted in the absence of any commercial or financial relationships that could be construed as a potential conflict of interest.

## Publisher’s Note

All claims expressed in this article are solely those of the authors and do not necessarily represent those of their affiliated organizations, or those of the publisher, the editors and the reviewers. Any product that may be evaluated in this article, or claim that may be made by its manufacturer, is not guaranteed or endorsed by the publisher.

## Footnotes

^1^ http://sgatools.ccbr.utoronto.ca/

^2^ https://www.yeastgenome.org/

^3^ https://yeastgfp.yeastgenome.org/

^4^ https://bioinfogp.cnb.csic.es/tools/venny/index.html

^5^ https://www.yeastgenome.org/

## Supplementary Material

Supplementary Table S1 Data of the three independent genome-wide screening.

Supplementary Table S2 List of genes for growth-deficient mutants under alkaline stress or saline stress generated by previous studies.

Supplementary Table S3 The transcriptome sequencing data under 40 or 0 mM NaHCO_3_ treatment.

Supplementary Table S4 List of significantly changed genes in the transcriptome under alkaline stress or saline stress yielded from previous studies.
